# Can titanium mesh influence local recurrence management after implant-based breast reconstruction?

**DOI:** 10.1186/s40064-015-1273-3

**Published:** 2015-09-04

**Authors:** Egidio Riggio, Camelia Chifu, Gabriele Martelli, Cristina Ferraris

**Affiliations:** Unit of Plastic and Reconstructive Surgery, Fondazione IRCCS Istituto Nazionale dei Tumori, Via Venezian 1, 20133 Milan, Italy; Unit of Breast Surgery, Fondazione IRCCS Istituto Nazionale dei Tumori, Via Venezian 1, 20133 Milan, Italy

**Keywords:** Nipple-sparing mastectomy, Implant reconstruction, Titanium-coated polypropylene mesh, Local recurrence risk, Granuloma

## Abstract

**Introduction:**

TiLOOP^®^ Bra is a permanent titanium-coated polypropylene mesh currently used in post-mastectomy breast reconstruction with implants. This mesh is generally presented as inducing low-grade inflammatory reactions, but only few reports focused on its possible side effects. In the case described here, the use of the mesh led to minor clinical problems that needed to be clinically and surgically managed at the same time as a local relapse.

**Case description:**

A patient with high-grade ductal carcinoma in situ underwent primary surgery (nipple-sparing mastectomy and one-stage reconstruction using the TiLOOP^®^ Bra mesh) and was subsequently referred for radiological and clinical investigation when various nodules became apparent during a follow-up physical examination. Prior to the histopathological proof, the diagnosis of local recurrence was complicated by the occurrence of an extensive granulomatous reaction in the fixation areas along with mild inflammatory changes scattered on the surface of the mesh.

**Discussion and evaluation:**

This case illustrates a side effect of titanium-coated permanent mesh in immediate implant-based reconstruction, i.e. the formation of granulomas in the inframammary fold, probably in the area where the mesh had been folded or fixed. We propose a safer technical approach to avoid the problem and a clinical management strategy for patients at high risk of local recurrence who develop granuloma-like nodules.

**Conclusions:**

A surgical technique is suggested to prevent granuloma formation. If, however, subcutaneous nodules that may be local recurrences do appear, they should not be interpreted by default as a granulomatous reaction, but should be fully investigated and possibly excised.

## Introduction

In recent years, manufacturers have created new products for breast reconstruction, some of them made of expensive biological materials, such as the acellular dermal matrix meshes, and others consisting of more affordable synthetic materials such as the titanium-coated polypropylene mesh. This mesh is increasingly used in Europe because of its satisfactory quality and cost-effectiveness and is generally presented as inducing low-grade inflammatory reactions, but only few reports have discussed its performance.

This case report describes the clinical problems that occurred in the treatment of a young patient affected by ductal carcinoma in situ (DCIS), who after a nipple-sparing mastectomy underwent immediate implant-based reconstruction using a TiLOOP^®^ Bra mesh (pfm medical, Cologne, Germany).

## Case description

In January 2012, a 27-year-old patient presented with a palpable mass of micronodules in the upper-outer quadrant of the right breast. Two years previously, she had undergone bilateral breast augmentation by subglandular access with a round Allergan CML implant (265 cc). No noteworthy medical conditions, risk factors or familial breast cancer history were reported.

A focused ultrasound scan found an area of increased glandular density while mammography showed 15 mm microcalcifications in the suspicious area. Breast MRI confirmed pathological contrast enhancement. The lesion was diagnosed as high-grade DCIS by histological examination after vacuum-assisted biopsy. Nipple-sparing mastectomy with sentinel lymph node biopsy and immediate implant-based reconstruction were performed in March 2012.

The patient’s subcutaneous layer was very thin and post-mastectomy skin flaps were less than 5 mm. The pectoralis major muscle was elevated by dividing the lower and medial insertions, and a TiLOOP^®^ Bra mesh was placed inferiorly and laterally in order to complete the pocket. The lower edge was fixed along the inframammary fold with single absorbable 3-0 sutures. The pathological diagnosis confirmed high-grade comedo-type DCIS (ER-positive, PgR-positive, Her2-negative). The nipple and sentinel lymph node biopsies were negative. No adjuvant therapies were recommended.

**Fig. 1:**
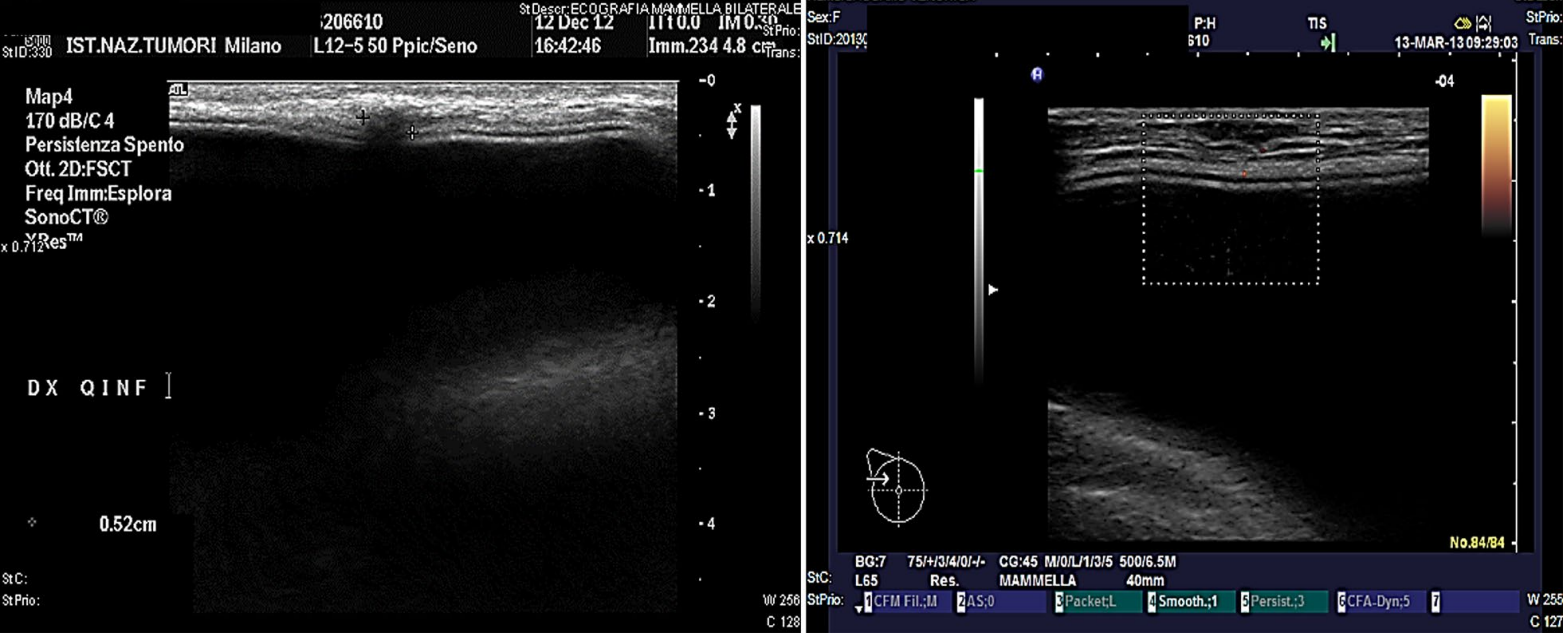
Ultrasound images. *Left* December 2012—the single nodule suggestive of granuloma in the lower-inner quadrant. *Right* March 2013—the nodule in the upper-outer quadrant, positive for recurrent DCIS

**Fig. 2:**
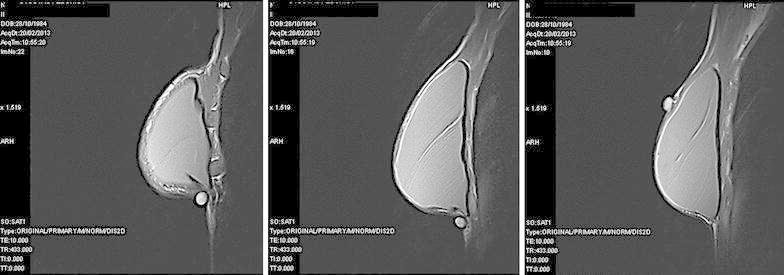
MRI images, February 2013*. Left* the first nodule (suggestive of granuloma) in the lower-inner quadrant. *Center* the nodule at the inframammary fold, at a distance of 2 cm from the first nodule. *Right* the nodule in the upper-outer quadrant (suggestive of breast cancer recurrence)

After a mastectomy, we schedule physical examination and breast ultrasound every 6 months and mammography once a year. MRI is used for patients with unclear ultrasound or mammography, with a family history of breast cancer or BRCA 1/2 mutation. In the present case, during a follow-up examination in December 2012 a palpable nodule was detected in the parasternal inframammary crease. Ultrasonography showed a 5-mm hypoechoic nodule consistent with granuloma, together with reactive axillary lymph nodes (Fig. 1, left). MRI in addition showed small areas of non-specific enhancement over the implant, particularly in the subcutaneous layer of the lower-inner quadrant, but did not reveal any distinct nodules. An MRI scan was planned 8 weeks later and clinical examination 12 weeks later. In February 2013, MRI showed decreased enhancement over the implant and the development of a new 8 × 3 mm area of homogeneous contrast enhancement in the upper-outer right quadrant, the site of the primary tumor. It also showed another nodule (<5 mm) in the lower-inner quadrant, at a distance of 2 cm from the first nodule (Fig. 2). After a focused ultrasound scan (Fig. 1, right), a wide excision of the upper-outer skin was performed in March and revealed a 2-cm multifocal comedo-type DCIS having the same biological profile as the primary tumor: ER-positive, PgR-positive, Her2 negative. In agreement with the plastic surgeon, the 5-mm- and <5 mm nodules in the inframammary area were not removed at the time, because they were suggestive of granuloma.

**Fig. 3:**
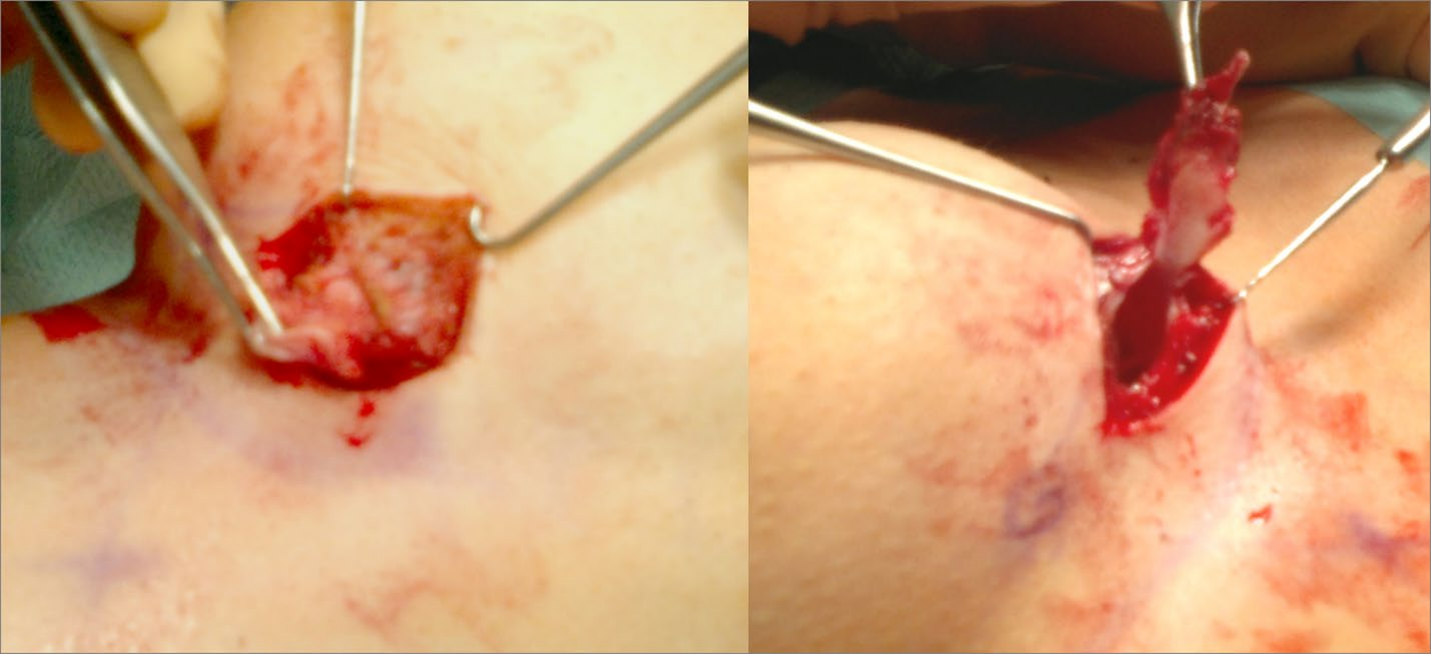
*Left* after the skin removal, the harvest of the outer fibrous layer above the tissue-integrated titanium mesh. *Right* the harvest of the inner layer composed of capsular tissue and tissue-integrated mesh layer

**Fig. 4:**
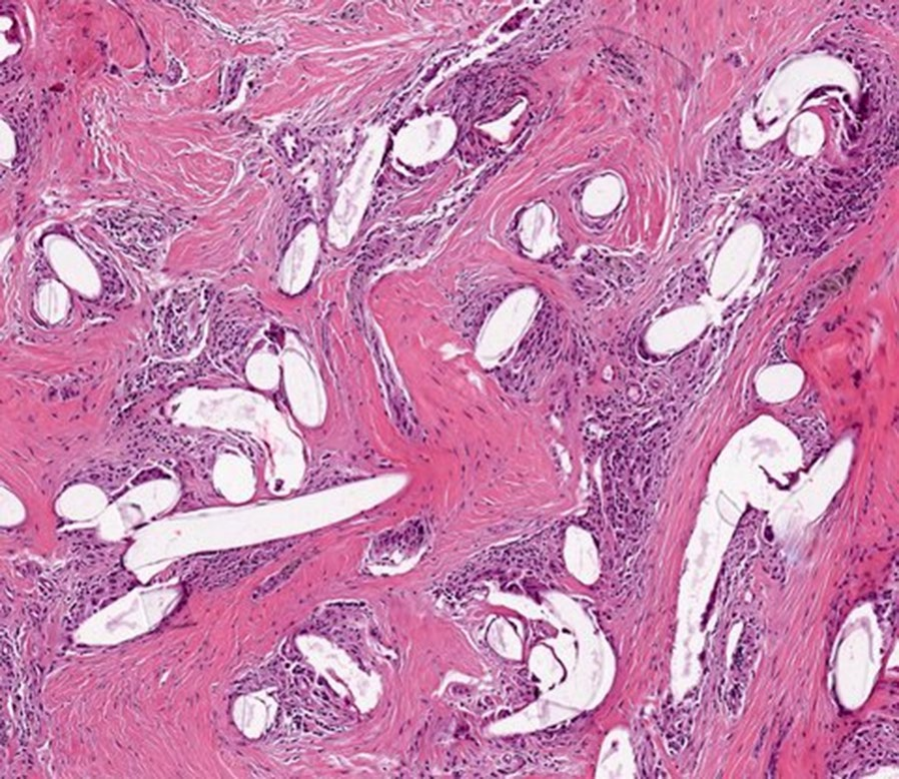
Histological features of the first nodule suggestive of granuloma, 14 months after implantation of the TiLOOP^®^ Bra mesh. Paraffin section of the subcutaneous tissue specimen, magnification ×10, H&E staining: mild infiltration with inflammatory cells and extensive granulomatous reaction with giant cells

Afterwards, a PET–CT scan showed minor uptake suggesting the presence of residual pathological tissue within the same upper-outer area over the implant. No pathological uptake was observed in the axillary lymph nodes or other organs. Although ultrasound imaging suggested granuloma as the initial diagnostic hypothesis, the concurrence with confirmed local recurrence, the limitations of PET–CT diagnostic resolution (5 mm), and the infiltration by multiple small nodules prompted a further interventional procedure. In April 2013 further surgery was performed to expand the soft-tissue resection in the upper-outer quadrant and remove the two suspicious palpable nodules in the medial submammary area. The capsular and inner mesh envelope were included in the excision (Fig. 3). The frozen sections showed fibrosis and chronic inflammation with granulomatous reaction (Fig. 4).

## Discussion and evaluation

Our case report discusses two issues: first a biological one, i.e. the development of a chronic granulomatous reaction, its prevalence, and how to avoid it; secondly, an oncological issue, i.e. the challenge posed by the development of multiple nodules in a context of high risk or actual occurrence of local relapse.

TiLOOP^®^ Bra is a titanium-coated lightweight polypropylene mesh, approximately 0.2 mm thick and with good biocompatibility. The product induces a light inflammatory reaction and no side effect have been reported so far.

The proliferation of inflammatory cells and giant cells at the fixation points observed in our case could have been triggered by the relatively thick layers of folded mesh. From 2012 to 2014, we performed about 50 implant reconstructions with TiLOOP^®^ Bra. Apart from the present case, only one case was observed where a granulomatous nodule was located in the inner quadrant, but MRI ruled out the possibility of local recurrence. This amounts to an incidence of 4 %, which is not low. But even if this side effect may be sporadic and not significantly interfere with the follow-up of breast cancer patients it is important to be aware of its possibility.

**Fig. 5:**
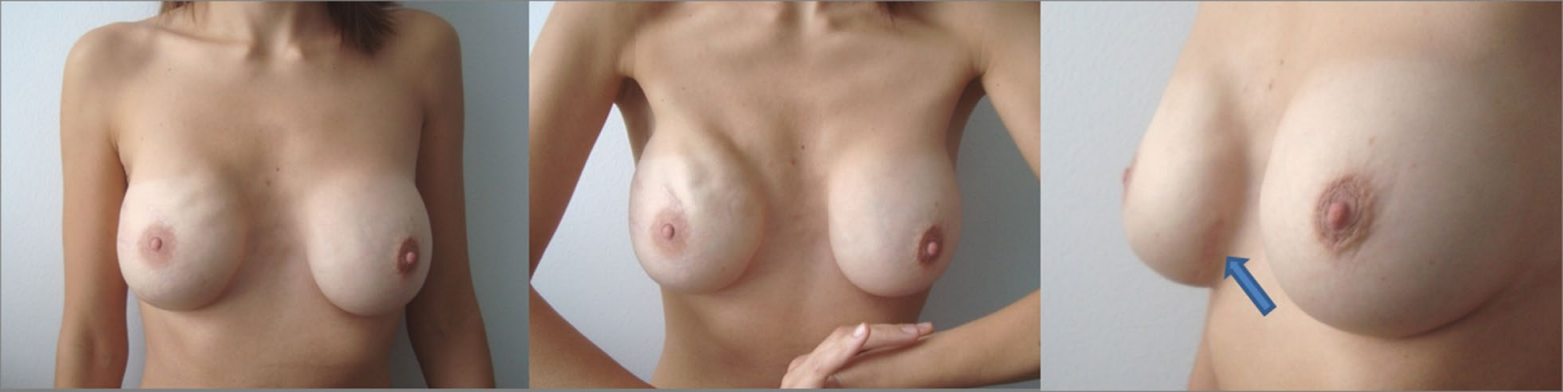
The patient after the excisional biopsies. The arrow shows the excision site of the TiLOOP^®^ Bra granuloma. One-stage reconstruction of the right breast was performed 19 months before (2012) with an Allergan anatomical and extra-projected implant, 410 FX 360 g. In the left breast, a subglandular Allergan CML round 265 cc implant was still present. Capsular reaction was grade 1 according to Baker’s classification on both sides. Note the different impact of rippling in the right breast due to the minimal capsular contracture of the implant coverage and the skin and muscle atrophy in the upper pole

The occurrence of mesh-related granuloma can be more easily detected in patients with extremely thin skin flaps. Among the few reports that have been published on titanium meshes, fewer still discussed the thickness of the mastectomy flaps and/or the technique used in the fixation of the mesh. Dieterich et al. (2013) argue that the mesh is minimally palpable and causes no discomfort, even when the mastectomy flaps are very thin. In general, the thickness of mastectomy flaps does not influence the success of the implant performed using TiLOOP^®^ Bra mesh. Nevertheless, in the absence of subcutaneous tissue, biological meshes (acellular dermal matrix) are preferred to TiLOOP^®^ Bra, which might explain the lack of reports on complications in patients with very thin flaps. In a paper that does not report any granuloma formation, Riggio et al. describe how the extreme pliability of the titanium mesh can also indirectly generate rippling, as happened to a patient with bilateral direct-to-implant reconstruction in the breast reconstruction where the mesh was used as opposed to the other breast reconstruction performed without mesh (Riggio et al. 2013). Flaps of thin to medium thickness cannot completely conceal the presence of the TiLOOP^®^ Bra, which can betray itself by dots and/or wrinkles. The titanium cover itself should induce minimal capsular reaction. In the case reported here, the three midline dots of the implant were almost visible between the lightweight mesh and the skin due to the thinness of the subcutaneous layer. This was a complication, however minor, from the surgeon’s point of view, even if in general the issue does not influence patients’ aesthetic appreciation of the result, as previously reported by several authors (Riggio et al. 2013; Dieterich et al. 2012). Also in this case the patient was entirely satisfied with the aesthetic result of surgery (Fig. 5).

To avoid the risk of granuloma formation after immediate breast reconstruction, we advise oncoplastic surgeons to fold the lowermost portion of TiLOOP^®^ Bra underneath the posterior part of the implant, without any wrinkling and with no fixation sutures. If the mesh is folded to be stitched along the inframammary fold, it can occasionally stimulate extensive granulomatous reaction with giant cells, but in the most recent cases where we applied the approach with folding only, the complication has not been observed.

The oncology issue is more challenging. Subcutaneous nodules of different origin forming at the same time and with the same clinical features have uncertain differential diagnosis due to their small size. In our case, the concurrent detection of two granuloma-like nodules and one nodule which was in fact a local recurrence required significant changes in the scheduled surveillance examinations (ultrasound, MRI and PET were performed 2–3 months after the mastectomy). Just by promptly carrying out multiple radiological investigations, the oncological diagnosis was not delayed by the presence of granulomas, while the only drawback for the patient was the number of open biopsies.

In the last two decades the incidence of DCIS has increased from 2 % to 15–20 % mainly as a result of increased mammographic screenings (Bleicher 2013; Owen et al. 2013), which in turn caused a rise in the number of nipple-sparing mastectomies and immediate reconstructions performed using products such as the titanium-coated mesh. Breast surgeons should be particularly vigilant with patients at high risk of early local recurrence of DCIS and inform plastic surgeons before reconstructive surgery is performed, because complete resection of the breast parenchyma is virtually impossible and, as has been noted, even with meticulous dissection under the skin, and also after radical mastectomy (Dreadin et al. 2012).

In general, when using a permanent fine mesh like TiLOOP^®^ Bra, the possible formation of granulomas should be taken into account. If a granuloma should be suspected, we suggest that resection is advisable unless the granulomatous reaction is confirmed by ultrasound and MRI and occurs in sites compatible with mesh folding. In case of patients at high risk of local recurrence, preventive resection may be advisable.

## Conclusions

It is known that titanium-coated meshes induce mild inflammation but our case showed it also can induce late granulomatous reactions in vivo. The pliability and thinness of TiLOOP^®^ Bra is generally considered an advantage, but this may not be the case in all patients. Future clinical studies should investigate if the use of TiLOOP^®^ Bra is consistently associated with granulomatous reaction, or whether the reaction is associated with specific technical approaches or patient peculiarities. Meanwhile, if reconstruction is performed with a titanium-coated mesh, as a preventive measure we suggest avoiding mesh fixation and, in selected patients, we advise evaluating alternative techniques or products (biological substitutes, e.g. acellular dermal matrix). Finally, if nodules should appear and there is a risk of local recurrence, further clinical investigation is needed or possibly surgical excision of the nodules.
